# A phase II trial comparing pazopanib with doxorubicin as first-line treatment in elderly patients with metastatic or advanced soft tissue sarcoma (EPAZ): study protocol for a randomized controlled trial

**DOI:** 10.1186/s13063-016-1434-x

**Published:** 2016-07-07

**Authors:** Annika Karch, Armin Koch, Viktor Grünwald

**Affiliations:** Institute for Biostatistics, Hannover Medical School, Carl-Neuberg-Strasse 1, 30625 Hannover, Germany; Clinic for Hematology, Hemostasis, Oncology and Stem Cell Transplantation, Hannover Medical School, Carl-Neuberg-Strasse 1, 30625 Hannover, Germany

**Keywords:** Soft tissue sarcoma, Elderly, Doxorubicin, Pazopanib, Noninferiority

## Abstract

**Background:**

Anthracycline-based treatment remains the backbone of chemotherapy for nonresectable soft tissue sarcomas (STS). More than 30 % of patients with STS are aged 60 years or older, limiting the choice of treatment to single-agent approaches for this elderly population. Hematological toxicity is frequent during doxorubicin monotherapy, grade 4 neutropenia is reported in 34 %, with a febrile neutropenia rate of 9 % in STS. We assume that comorbidities in the elderly population may limit tolerability of doxorubicin, and novel agents may improve tolerability and health-related quality of life while maintaining efficacy. We therefore investigated whether the tyrosine kinase inhibitor pazopanib exerts such a clinical benefit in elderly patients with STS (pazopanib for elderly [the EPAZ study]).

**Methods/design:**

This study is a randomized, controlled, open-label, multicenter, phase II noninferiority trial in which pazopanib 800 mg once daily is being compared six cycles of intravenous doxorubicin 75 mg/m^2^ as first-line treatment in elderly patients (≥60 years) with metastatic or advanced STS. A total of 120 patients will be randomized 1:2 to receive doxorubicin or pazopanib, stratified by Eastern Cooperative Oncology Group performance status (0–1 vs. 2) and liposarcoma histology (yes vs. no).

The primary endpoint is progression-free survival based on local tumor assessment according to Response Evaluation Criteria in Solid Tumors criteria. Secondary endpoints include grade 4 neutropenia and febrile neutropenia in hierarchical order, as well as overall survival, objective response rate, health-related quality of life, and geriatric assessments.

**Discussion:**

Pazopanib is associated with promising tolerability according to previous studies and may offer a significant clinical advantage in first-line treatment of STS compared with doxorubicin. The elderly population seems especially appealing for such an approach, since these patients are not suitable for aggressive combination therapy. The EPAZ study will confirm whether pazopanib may be an alternative to toxic chemotherapy for elderly patients with STS.

**Trial registration:**

ClinicalTrials.gov NCT01861951; registered on 11 April 2013. EudraCT 2011-004168-30; registered on 4 June 2012.

**Electronic supplementary material:**

The online version of this article (doi:10.1186/s13063-016-1434-x) contains supplementary material, which is available to authorized users.

## Background

Soft tissue sarcomas (STS) are malignant, heterogeneous tumors that typically arise in the mesodermal tissues of the extremities (50 %), trunk and retroperitoneum (40 %), or head and neck (10 %) [1]. Their histology is diverse, and more than 50 different subtypes are recognized in the current classification system [2].

STS in adults are rare and have an estimated incidence of 4–5 per 100,000 per year in Europe [3]. In the United States, more than 10,000 new cases were reported in 2010 [4]. The aggressiveness of STS is emphasized by the high death rate, with nearly 4000 deaths occurring in the United States in 2010. Risk factors for recurrent disease are described as incomplete resection, high-grade histology, STS larger than 5 cm, and deep location [5].

As a result of STS heterogeneity, the development of an effective antitumor agent has been difficult. For decades, doxorubicin has formed the backbone of systemic treatment of a wide range of cancers, including hematological malignancies, many types of carcinoma, and unresectable or metastatic STS [6]. Doxorubicin treatment is frequently associated with hematological toxicity. Grade 4 neutropenia has been reported in 34–37 % of patients with STS during treatment with doxorubicin, and 9–13 % of all patients being treated with doxorubicin experience febrile neutropenia [7, 8].

Elderly patients are known to be prone to comorbidities, and aggressive treatment may not be feasible for these patients, which is why multiagent chemotherapy is not recommended for them. However, grade 4 neutropenia and neutropenic fever are considered severe side effects of palliative treatment and may be poorly tolerated by elderly patients. Finding an alternative agent with similar efficacy but fewer and less severe adverse effects (AEs) is therefore particularly important for this patient group.

A promising candidate for this purpose could be pazopanib, which is an orally administered, potent, multitargeted receptor tyrosine kinase inhibitor. Pazopanib has demonstrated encouraging results in clinical trials in renal cell carcinoma, ovarian cancer, early-stage non-small cell lung cancer, breast cancer, and cervical cancer [9–14]. In a phase II study of patients with advanced or metastatic STS receiving pazopanib as a second-line therapy, the progression-free rate (PFR) at 12 weeks was 44 % (18 of 41 subjects) for leiomyosarcoma, 49 % (18 of 37 subjects) for synovial sarcoma, 26 % (5 of 19) for adipocytic sarcoma, and 39 % (16 of 41 subjects) for other types of sarcoma [15]. Agents with a PFR of at least 40 % at 12 weeks are suggested to show adequate drug activity in pretreated patients [16]. Therefore, further clinical development of pazopanib excluded patients with adipocytic sarcoma from clinical trials after the given phase II results. The PALETTE (pazopanib for metastatic soft-tissue sarcoma) study investigators assessed pazopanib in comparison with placebo in STS excluding adipocytic differentiation [17]. Pazopanib achieved a significant improvement in median progression-free survival (PFS) from 1.6 to 4.6 months (*p* < 0.0001), which was the primary endpoint of the trial. Overall survival increased from 10.7 to 12.5 months (HR 0.86, 95 % CI 0.7–1.1; *p* = 0.25), but the study size was too small to detect such a treatment effect.

Overall, pazopanib offers a distinct mechanism of action and spectrum of adverse events, thereby offering some advantage over conventional chemotherapy. In our present study, we are testing this hypothesis in comparison with single-agent doxorubicin in elderly patients with STS.

## Methods

### Study objectives

The primary aim of this study is to show that PFS in the pazopanib group is not inferior to PFS in the doxorubicin group. Key secondary objectives are to show superiority of pazopanib compared with doxorubicin regarding neutropenia grade 4 and/or febrile neutropenia. Further secondary objectives are to analyze treatment effects on overall survival, objective tumor response, time to onset of response, quality of life, safety, and tolerability, as well as to investigate predictive biomarkers.

### Study design

This trial is a randomized, open-label, multicenter, phase II noninferiority study designed to compare pazopanib with doxorubicin as first-line treatment in elderly patients with metastatic or advanced STS. A total of 120 patients from 13 (German and Belgian) study centers will be enrolled, comprising 40 patients in the doxorubicin arm and 80 patients who will receive pazopanib.

The overall study duration will be approximately 50 months, including a recruitment period of 36 months, a 4.5-month period of treatment with doxorubicin (18 weeks) or pazopanib until treatment failure, and follow-up of at least 10 months.

**Fig. 1:**
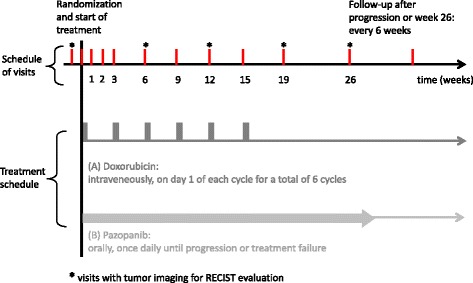
Schedule of visits and treatment. *RECIST* Response Evaluation Criteria in Solid Tumors

Baseline and follow-up assessments are performed according to a predefined schedule (see Fig. 1 and Additional file 1: Figure S1). Follow-up visits occur at the same time points for both treatment arms. In both treatment groups, an end-of-treatment visit 4 weeks after the end of treatment is performed. Tumor images obtained by performing computed tomography or magnetic resonance imaging for evaluation of response and progress are taken every 6–7 weeks in the first 6 months and every 12 weeks after week 26 until progression. The Standard Protocol Items: Recommendations for Interventional Trials (SPIRIT) checklist and assessment figure of the trial can be found in Additional file 2: Table S1 and Additional file 1: Figure S1.

### Ethical considerations

The trial protocol was approved by the ethics committee of Hannover Medical School and the ethics committees of all participating study centers (see Additional file 3). The study is registered at ClinicalTrials.gov (identifier NCT01861951) and ClinicalTrialsRegister.eu (identifier 2011-004168-30). The results of the trial will be reported in accordance with the Consolidated Standards of Reporting Trials (CONSORT) statement [18, 19].

### Study population

Male and female patients with metastatic or advanced STS who are at least 60 years of age are screened for the study. After they provide signed written informed consent, their eligibility is determined on the basis of the inclusion and exclusion criteria listed in Table 1.

**Table 1:** Inclusion and exclusion criteria

Inclusion criteria	Exclusion criteria
• Signed written informed consent	• STS of uncertain differentiation (epithelioid, alveolar soft part, clear cell, desmoplastic small round cell, malignant mesenchymoma, PEComa), chondrosarcoma, Ewing sarcoma/PNET, chordoma, malignant solitary fibrous tumors, embryonal rhabdomyosarcoma, osteosarcoma, gastrointestinal stromal tumors, dermatofibrosarcoma protuberans, inflammatory myofibroblastic sarcoma (low grade), neuroblastoma, malignant mesothelioma, mixed mesodermal tumors of the uterus
• Age ≥60 years	• Prior malignancy, except for subjects who have been disease-free for 2 years, or complete resection of nonmelanomatous skin carcinoma, or successfully treated in situ carcinoma or incidental prostate cancer (TNM stage T1a or T1b)
• ECOG performance status of 0–2	• History or clinical evidence of CNS metastases; previously treated subjects without signs of activity are allowed
• Histologically confirmed diagnosis of metastatic or advanced STS of intermediate or high grade	• Clinically significant gastrointestinal abnormalities that may increase the risk for gastrointestinal bleeding or may affect absorption of IMP
• Evidence of progressive disease within 6 months prior to study inclusion	• Presence of uncontrolled infection
• Availability of archived tumor tissue of the most recent histology	• QTc >480 milliseconds using Bazett’s formula
• Adequate organ system function as determined by laboratory assessment	• History of any of the following cardiovascular conditions within the past 6 months: cardiac angioplasty or stenting, myocardial infarction, unstable angina, coronary artery bypass graft surgery, symptomatic peripheral vascular disease, cerebrovascular accident (including TIA), pulmonary embolism, or untreated deep vein thrombosis
• Adequate contraception for patients or partners with childbearing potential	• Class III or IV congestive heart failure as defined by the NYHA classification system
• Negative pregnancy test for women of childbearing potential	• Poorly controlled hypertension
	• Major surgery or trauma within 28 days before first dose of IMP and/or presence of any nonhealing wound, fracture, or ulcer
	• Evidence of active bleeding or bleeding diathesis
	• Known endobronchial lesions and/or lesions infiltrating major pulmonary vessels
	• Hemoptysis in excess of 2.5 ml once within 8 weeks of first dose of IMP
	• Any serious and/or unstable preexisting medical, psychiatric, or other condition that could interfere with subject’s safety, provision of informed consent, or compliance with study procedures
	• Unable or unwilling to discontinue use of prohibited medications for at least 14 days or 5 half-lives of a drug (whichever is longer) prior to the first dose of IMP and for the duration of the study
	• Treatment with any anticancer therapies
	• Any ongoing toxicity from prior anticancer therapy that is higher than CTCAE grade 1 and/or that is progressing in severity, except alopecia
	• Prior systemic therapy for metastatic or advanced disease; neoadjuvant or adjuvant chemotherapy is allowed, unless disease progression occurred within 6 months following end of treatment
	• Participation in any other clinical trial within 30 days before the study begins
	• Known hypersensitivity to any component of IMPs

*Abbreviations: CTCAE* Common Terminology Criteria for Adverse Events, *ECOG* Eastern Cooperative Oncology Group, *IMP* investigational medical product, *NYHA* New York Heart Association, *PEComa* perivascular epithelioid cell tumor, *PNET* primitive neuroectodermal tumor, *QTc* corrected QT interval, *STS* soft tissue sarcomas, *TIA* transient ischemic attack

### Randomization and blinding

This is an open-label study with a 1:2 randomization ratio for doxorubicin/pazopanib. Blinding was considered to be ethically infeasible due to the different dosing schedules and routes of administration. A fax randomization is centrally performed at the Institute for Biostatistics in Hannover according to a permuted block randomization list with varying block sizes. Randomization is stratified for Eastern Cooperative Oncology Group (ECOG) performance status (0–1 vs. 2) and liposarcoma histology (yes vs. no) at randomization.

### Interventions

#### Control intervention

Doxorubicin is an anthracycline antibiotic with a mechanism of action aimed at topoisomerase inhibition. Doxorubicin (monotherapy) is administered intravenously every 3 weeks at a dose of 75 mg/m^2^ body surface area for a total of six cycles.

#### Experimental intervention

Pazopanib is a once-daily, orally administered angiogenesis inhibitor targeting vascular endothelial growth factor (VEGF), platelet-derived growth factor, and c-kit receptors [20]. By inhibiting these receptors, pazopanib may stop or slow the rate of tumor growth and development. Pazopanib is provided as white tablets with two doses at 200 mg and 400 mg. Patients receive a daily dose of 800 mg. Treatment is administered until disease progression, treatment failure, or death due to any cause, whichever occurs first. Pazopanib has been approved by the U.S. Food and Drug Administration, the European Medicines Agency, and other regulatory authorities as a monotherapy for patients with advanced renal cell carcinoma and as second-line therapy for advanced STS.

#### Dose adjustments

Dose reduction, delay of administration, and treatment interruption might become necessary for reasons of toxicity in both treatment groups. Patients with dose adjustments are closely monitored (weekly). Dose modification algorithms are given in the study protocol. As a general rule, pazopanib is reduced stepwise each 10–14 days in 200-mg decrements. If the toxicity has abated with dose reduction and dose re-escalation is considered safe by the investigator, the pazopanib dose is increased stepwise back to the pre-event dose. For doxorubicin, a 1-week treatment delay and/or a 20 % dose reduction (i.e., 60 mg/m^2^) are suggested. Dose modifications to 60 mg/m^2^ doxorubicin are allowed once and cannot be re-escalated. If toxicity does not abate during the monitoring period, administration of pazopanib or doxorubicin is interrupted and/or the dose is further reduced. It should be permanently discontinued for any hematological or nonhematological toxicity requiring an interruption of ≥14 days.

### Outcomes

#### Primary endpoint

The primary endpoint is PFS, calculated as time from the date of randomization until the date of first objective documentation of disease progression, treatment failure, or death due to any cause, whichever occurs first.

#### Secondary endpoints

Key secondary outcomes are the rates of grade 4 neutropenia and of febrile neutropenia. Rates are defined as the number of scheduled examinations where grade 4 neutropenia is diagnosed (febrile neutropenia respectively) divided by the follow-up time until progression, death, or the end of the study, or (in case of treatment switch) until initiation of another anticancer treatment. Incident diagnoses will be considered at the next examination. In addition to the primary outcome, PFRs after 12 and 26 weeks from the date of randomization and overall survival, defined as time from randomization to death due to any cause, will be estimated. Further secondary endpoints are objective tumor response, calculated as objective response rate, along with time to onset of response. Assessment of response and progression is based on Response Evaluation Criteria in Solid Tumors version 1.1, without independent review of tumor response or progression events.

Safety and tolerability will be assessed continuously throughout the course of the study. Toxic and side effects are evaluated by using the National Cancer Institute Common Terminology Criteria for Adverse Events version 4.0. Quality of life is measured with the European Organisation for Research and Treatment of Cancer (EORTC) 30-item core quality of life questionnaire (the QLQ-C30). Geriatric assessments are made according to the EORTC Elderly Task Force guidelines.

The predictive role of biomarkers will be assessed to predict the PFS rate at 26 weeks after the start of treatment. Biomarkers measured in the study will include placental growth factor, fibroblast growth factor, VEGF, PDGF, angiopoietin 2, and interleukin 8. Blood samples will be taken at baseline, at 2 weeks, and at each scheduled follow-up visit starting from 3 weeks after randomization.

#### Further assessments

A large panel of clinical and laboratory data is being collected in the trial. At each visit, a physical examination is conducted, including assessment of vital signs, and information on hematology and clinical chemistry is obtained, which are particularly important for assessment of tolerability. Evaluation furthermore comprises liver function tests, coagulation tests, proteinuria via urinalysis, thyroid function tests, and lipid tests. Echocardiography is performed at baseline and at week 26. Based on the key secondary endpoints, primary and secondary prophylaxis of neutropenia assessment is of key importance. Primary and secondary prophylaxis of neutropenia are routinely assessed and documented for each cycle. The participating trial centers have decided at baseline whether they will implement primary prophylaxis.

### Sample size considerations

Sample size is feasibility-driven because of orphan drug conditions [21], and, in consequence, our power calculation describes under which circumstances this trial can reach an adequate power for an achievable sample size. In this study a maximum of 120 patients can be included within a reasonable time frame of 3 years of recruitment. With a 1:2 randomization, this leads to 40 patients in the doxorubicin arm and 80 patients in the pazopanib arm.

The one-sided type I error rate is set to 2.5 %. The treatment groups will be compared by using HRs. For both treatment groups, a median PFS of 6 months is assumed [8, 22]. The accrual time is 36 months, and the minimum observational time is 10 months. Under these conditions, noninferiority with a margin of 1.8 for the HR can be concluded with a power of >80 %. An anticipated rate of dropout and exclusion from the primary analysis population of 10 % maintains a power of 79–80 %.

### Statistical analysis

#### Primary analysis

The primary analysis will be performed on the per-protocol (PP) population and, as a sensitivity analysis, on the intention-to-treat (ITT) population. Consistency between results in ITT and PP analysis is needed to draw any conclusion regarding differences in PFS. The PP population will include all randomized patients who received the assigned study treatment and took the study medication according to the protocol (allowing for dose reduction and/or temporary interruption, or termination of trial drug intake without other anticancer therapy being initiated before documented progression). If patients start another anticancer therapy, they will be censored at the respective time point in the ITT analysis.

For PFS, a Cox regression model will be used to calculate the HR of pazopanib/doxorubicin and the respective two-sided 95 % CI. If the upper limit of this CI in the PP population is less than 1.8, noninferiority will be concluded. The Cox regression model is adjusted for ECOG score (0–1 vs. 2) and liposarcoma histology (yes vs. no). Kaplan-Meier curves will be drawn.

#### Secondary and safety analyses

If statistical significance is reached for the primary endpoint, a confirmatory analysis of the key secondary endpoints grade 4 neutropenia and febrile neutropenia will be performed in hierarchical order. If no statistically significant difference for grade 4 neutropenia is observed, febrile neutropenia will be considered descriptively. The rates of grade 4 neutropenia will be analyzed with a Poisson regression model adjusted for ECOG score and liposarcoma histology and allowing for clustering of the outcome within patients by using a random effects term. Superiority of pazopanib will be concluded if the upper boundary of the two-sided 95 % CI for the rate ratio (pazopanib/doxorubicin) is below 1. Rates of febrile neutropenia will be analyzed with the same approach. Due to the hierarchical order in confirmatory testing, no adjustment for multiplicity has to be done.

For the analysis of overall survival, Kaplan-Meier curves and Cox regression will be used. For analysis of PFRs after 12 and 26 weeks, a logistic regression model will be used to calculate the OR between the two treatment groups. Corresponding two-sided 95 % CIs will be calculated. The objective tumor response rate will be computed with 95 % CIs in each treatment arm. Patients with progression, early death, or unknown status are considered as failures. Time to onset of response will be reported as median and range. Occurrence and frequency of AEs and serious AEs will be evaluated separately for both treatment groups, and frequency tables and corresponding 95 % CIs will be shown.

The key secondary analyses and analyses of all further efficacy endpoints will be conducted on the ITT population with sensitivity analyses based on the PP population. Analysis of safety endpoints will be conducted on the full analysis set. No interim analyses are planned in this trial.

## Discussion

Pazopanib has been approved as a monotherapy treatment for patients with advanced renal cell carcinoma and as second-line therapy for advanced STS. On the basis of current clinical data suggesting a favorable safety profile of pazopanib, it is justifiable to develop pazopanib for use as first-line treatment for STS. The elderly population seems especially appealing for such an approach, since these patients generally are not suitable for aggressive combination therapy, and thus pazopanib could be an alternative to toxic chemotherapy. For this target population, the EPAZ trial (A Trial Comparing Two Medications as First Treatment in Elderly Patients with Metastatic or Advanced Soft Tissue Sarcoma) will provide important insights on the efficacy and safety of pazopanib compared with the current standard treatment with doxorubicin.

The design and performance of a randomized controlled trial in elderly patients with STS are hampered by the facts that STS is a rare disease with orphan status [21] and only a small number of patients are available for study enrollment. According to the respective guidelines [23, 24], clinical trials in small populations ought to be planned with the best, most suitable study design to produce unbiased and interpretable study results. Hence, a randomized, multicenter, active control, noninferiority trial design with PFS as a valid primary surrogate endpoint has been chosen.

We extensively discussed the noninferiority margin prior to the study. A margin of 1.8 for the HR of disease progression appears to be quite high compared with noninferiority margins of other cancer studies (HR 1.2–1.5). The chosen margin of HR of 1.8 corresponds to a possible decrease in PFS from 6 months to 3.3 months, which, from a clinical point of view, is relevant and unacceptable. However, in view of the small achievable sample size, the study will inevitably generate large CIs for the HR and will therefore be severely underpowered when using the usual “resolution.” There are different strategies to handle this problem: (1) increase the significance level to obtain tiny CIs or (2) relax the noninferiority margin, or (3) change nothing and be prepared to obtain nonsignificant (but clinically interpretable) results. We decided to customize the noninferiority margin to a level at which the study has appropriate power to be formally successful if both treatments have at least the same PFS (HR ≤1). We should keep in mind that the power diminishes rapidly if the true HR is only slightly greater than 1 (i.e., in favor of doxorubicin). In this study setting of rare patients and very wide CIs, a strong focus can be put only on the observed point estimates and their clinical interpretation. Decisions regarding application of the proposed treatment strategy or further clinical studies will not be based solely on the significance or nonsignificance of a defined primary endpoint, but they will be made taking into account all relevant aspects in the entire trial dataset. This means that all benefits and risks observed in the EPAZ trial will be discussed in detail and will form the basis of an overall clinical conclusion.

We included assessment of key secondary endpoints that are clinically relevant. Neutropenia and febrile neutropenia are believed to have high impact in the elderly population. Therefore, both factors were considered relevant in the context of the noninferiority design with broad margins. Nevertheless, observed point estimates and confidence limits of PFS rates and of the respective HR (pazopanib/doxorubicin) will particularly be assessed for clinically relevant differences and will be carefully discussed when interpreting the results.

Randomization and analysis are stratified for two important prognostic factors: ECOG performance status and liposarcoma histology. It can be assumed that the power is increased by performing stratified analyses [23]. Nonetheless, a limitation of the EPAZ study design is the low power for drawing an overall positive conclusion. Obviously, the aim of the study is to show comparable efficacy accompanied by considerable advantages in terms of safety issues and increased health-related quality of life (HRQoL) as a consequence. However, the study is powered only for the primary objective, because the orphan conditions do not allow us to adequately power the study for the primary as well as the two key secondary endpoints, or even additional secondary objectives. We expect that, on basis of the safety profile of either drug and the continuous evaluation of HRQoL, we will be able to show a clinically meaningful benefit of the given therapy. To our understanding, EPAZ is the first study in STS that weights the quality of PFS and will provide data regarding comorbidities and HRQoL in the elderly population.

Pazopanib is an effective treatment in STS. Its activity in previously treated patients is within the range of contemporary first-line activity of doxorubicin [8, 17]. However, the classes of agents used differ substantially between pazopanib and doxorubicin. While pazopanib is an oral tyrosine kinase inhibitor that inhibits the VEGFR, doxorubicin is a classical chemotherapeutic compound. Hence, its toxicity profile differs substantially. It is not known how continuous exposure with chronic toxicity affects patients in comparison with chemotherapy, with its cyclic treatment and adverse events. The principal applicability to elderly patients has been shown in metastatic renal cancer, a disease whose incidence peaks in the sixth and seventh decades of life [25]. If both noninferiority in efficacy and an improved tolerability of pazopanib (and thus improved quality of life) are verified in our trial, a new first-line treatment strategy for elderly patients with STS may become available.

## Trial status

This trial was initiated in October 2012. Recruitment was completed in March 2016, and follow-up is ongoing.

## Abbreviations

AE, adverse effect; CONSORT, Consolidated Standards of Reporting Trials; CTCAE, Common Terminology Criteria for Adverse Events; ECOG, Eastern Cooperative Oncology Group; EORTC, European Organisation for Research and Treatment of Cancer; EPAZ, A Trial Comparing Two Medications as First Treatment in Elderly Patients With Metastatic or Advanced Soft Tissue Sarcoma; HRQoL, health-related quality of life; IMP, investigational medical product; ITT, intention to treat; NYHA, New York Heart Association; PDGF, platelet-derived growth factor; PEComa, perivascular epithelioid cell tumor; PFR, progression-free rate; PFS, progression-free survival; PNET, primitive neuroectodermal tumor; PP, per protocol; QLQ-C30, European Organisation for Research and Treatment of Cancer 30-item core quality of life questionnaire; QTc, corrected QT interval; RECIST, Response Evaluation Criteria in Solid Tumors; STS, soft tissue sarcomas; TIA, transient ischemic attack; VEGF, vascular endothelial growth factor

## Additional files

Additional file 1: Figure S1. SPIRIT schedule of enrollment, assessments, and interventions in the EPAZ study. (DOC 96 kb) Additional file 2: Table S1. SPIRIT 2013 Checklist: Recommended items to address in a clinical trial protocol and related documents. (PDF 1880 kb) Additional file 3: List of all ethical bodies that approved the EPAZ study. (DOCX 14 kb)
