# Supplementary material for: A phase II trial comparing pazopanib with doxorubicin as first-line treatment in elderly patients with metastatic or advanced soft tissue sarcoma (EPAZ): study protocol for a randomized controlled trial
Source: Trials. 2016 Jul 7;17:312. doi: 10.1186/s13063-016-1434-x (PMC4936293; doi:10.1186/s13063-016-1434-x)
Supplement: Additional file 1: Figure S1. — SPIRIT schedule of enrollment, assessments, and interventions in the EPAZ study. (DOC 96 kb) [file 13063_2016_1434_MOESM1_ESM.doc]

**Additional file 1: Figure A1: SPIRIT s**chedule of enrolment, assessments and interventions in the EPAZ study.

|  | **Baseline** | **Core Study** | | | | | | | | | | **Extension study** | | **EOT ^n^** | **Follow-up** |
| --- | --- | --- | --- | --- | --- | --- | --- | --- | --- | --- | --- | --- | --- | --- | --- |
| **Week** | wk-3 to 0 | 0^$^ | 1 | 2 | 3 | 6 | 9 | 12 | 15 | 19 | 26 | q6wks | q12wks | 4 wks after last dose | q12wks |
| **Informed consent** | **X** |  |  |  |  |  |  |  |  |  |  |  |  |  |  |
| **Randomization** |  | **X** |  |  |  |  |  |  |  |  |  |  |  |  |  |
| **Medical history** | **X** |  |  |  |  |  |  |  |  |  |  |  |  |  |  |
| **Adverse events,**  **concomitant medication** | **X** | **continuously during study until end of treatment (EOT)** | | | | | | | | | | **X** |  | **X** |  |
| **Physical examination** | **X** | **X** |  |  | **X** | **X** | **X** | **X** | **X** | **X** | **X** | **X** |  | **X** |  |
| **Vital signs ^a^** | **X** | **X** | **X** | **X** | **X** | **X** | **X** | **X** | **X** | **X** | **X** | **X** |  | **X** |  |
| **ECOG performance status** | **X** | **X** |  |  | **X** | **X** | **X** | **X** | **X** | **X** | **X** | **X** |  | **X** |  |
| **Pregnancy test (if applicable)** | **X** |  |  |  |  |  |  |  |  |  |  |  |  |  |  |
| **Chemistry ^b^** | **X** |  |  |  | **X** | **X** | **X** | **X** | **X** | **X** | **X** | **X** |  | **X** |  |
| **Liver function tests ^c^** | **X** |  |  | **X** | **X** | **X** | **X** | **X** | **X** | **X** | **X** | **X** |  | **X** |  |
| **Lipids ^d^** | **X** |  |  |  |  |  |  |  |  |  |  |  |  |  |  |
| **Thyroid function ^e^** | **X** |  |  |  |  | **X** |  | **X** |  | **X** | **X** | **X** |  | **X** |  |
| **Hematology ^f^** | **X** |  | **X** | **X** | **X** | **X** | **X** | **X** | **X** | **X** | **X** | **X** |  | **X** |  |
| **Coagulation test ^g^** | **X** |  |  |  | **X** | **X** | **X** | **X** | **X** | **X** | **X** |  | **X** | **X** |  |
| **Urinanalysis ^h^** | **X** |  |  |  | **X** | **X** | **X** | **X** | **X** | **X** | **X** | **X** |  | **X** |  |
| **12-lead ECG** ^i^ | **X** |  |  |  | **X** |  |  |  |  | **X** |  |  |  | **X** |  |
| **LVEF** | **X** |  |  |  |  |  |  |  |  |  | **X** |  |  |  |  |
| **CT/MRI of tumor lesions ^j^** | **X** |  |  |  |  | **X** |  | **X** |  | **X** | **X** |  | **X** |  |  |
| **Quality of life ^k^** | **X** | **X** |  |  | **X** | **X** | **X** | **X** | **X** | **X** | **X** |  | **X** | **X** |  |
| **Geriatric assessment ^l^** | **X** |  |  |  |  |  |  | **X** |  |  | **X** |  | **X** |  |  |
| **Biomarkers (serum)** | **X** |  |  | **X** | **X** | **X** | **X** | **X** | **X** | **X** | **X** | **X** |  | **X** |  |
| **Primary tumor blocks ^m^** | **X** |  |  |  |  |  |  |  |  |  |  |  |  |  |  |
| **Arm A: doxorubicin 75 mg/m2 iv, q3wk, injection** |  | **X** |  |  | **X** | **X** | **X** | **X** | **X** |  |  |  |  |  |  |
| **Arm B: pazopanib 800 mg OD, continuous** |  | **continuously during study until progression** | | | | | | | | | | | |  |  |
| **Information of survival and subsequent therapies** |  |  |  |  |  |  |  |  |  |  |  |  |  |  | **X** |
|  |  |  |  |  |  |  |  |  |  |  |  |  |  |  |  |
| ^$^ Baseline laboratory tests need to be repeated if start of treatment is >7 days from baseline assessment. | | | | | | | | | | | | | | | |
| ^a^ Vital signs include blood pressure (BP), heart rate, temperature, height, and weight. Monitoring of BP only: A measurement of BP is mandatory on day 1, 8, 15, and day 1 of every subsequent cycle. Further readings are required on day 1 of a given cycle. Additional BP monitoring is encouraged throughout the treatment based on the patient’s individual situation. BP can be assessed by any method (i.e. at home or by another physician) as long as the study physician is informed of the measurement, verifies any measurement that is not normal, and takes appropriate action. | | | | | | | | | | | | | | | |
| ^b^ Calcium, magnesium, potassium, sodium, inorganic phosphate, glucose, lactate dehydrogenase, albumin, urea, and creatinine. Assessments may be performed more frequently if clinically indicated. Estimated creatinine clearance will be calculated using the MDRD (modification of diet in renal disease) method. Alternatively, creatinine clearance may also be determined based upon cystatin c assessment. | | | | | | | | | | | | | | | |
| ^c^ AST, ALT, alkaline phosphatase, albumin, and bilirubin. A direct bilirubin level will be obtained only if the total bilirubin level is greater than 1.5 x upper limit of normal (ULN). Liver chemistry threshold stopping criteria and dose modification guidelines have been designed to ensure subject safety. | | | | | | | | | | | | | | | |
| ^d^ Cholesterol and triglycerides. Additional testing may be performed if clinically indicated. In such a case, the patient should be in a fasting state. | | | | | | | | | | | | | | | |
| ^e^ Thyroid function test to assess thyroid stimulating hormone (TSH) will be done every 6 weeks. In case of abnormal findings, measurement of free T3 and T4 are required. | | | | | | | | | | | | | | | |
| ^f^ Hematology is to be collected weekly until week 18 (i.e. the end of cycle 6) during core study. Hematology includes hemoglobin, hematocrit, red blood cell count, white blood cell count, lymphocytes, total neutrophils, and platelet count. Baseline hematology needs to be repeated if the interval between start of treatment and baseline assessment is >7 days. | | | | | | | | | | | | | | | |
| ^g^ Coagulation tests include PT/INR and aPTT. | | | | | | | | | | | | | | | |
| ^h^ Dipstick assessment with pH, protein, glucose, bilirubin, ketones, blood cells, and leukocytes. If ≥ 2+ proteinuria occurs by dipstick analysis, evaluation of the urine protein to creatinine ratio (UPC) is mandatory. If the UPC is ≥ 3, then the dose modification table guidelines should be followed. | | | | | | | | | | | | | | | |
| ^i^ ECG: In case of significant abnormal findings, ECG will be repeated every 8 weeks until its resolution or end of treatment, whichever comes first. | | | | | | | | | | | | | | | |
| ^j^ Tumor measurement: will be performed within 1 week prior to visit, i.e. last week of a given cycle for the first 3 CTs/MRIs. An additional scan will be performed at week 26 ± 7days for all patients without tumor progression. Thereafter, scans will be performed every 12 weeks. CTs and MRIs will be performed in the context of standard care. In patients with brain metastases at baseline, routine follow-up of CNS lesions should be performed every 12 weeks by CT or MRI. | | | | | | | | | | | | | | | |
| ^k^ QLQ-C30 | | | | | | | | | | | | | | | |
| ^l^ According to EORTC ETF. | | | | | | | | | | | | | | | |
| ^m^ If not available 8 pretreated representative unstained slides. | | | | | | | | | | | | | | | |
| ^n^ week 19 is EOT for the doxorubicin arm unless patients have progressed. | | | | | | | | | | | | | | | |
