# Supplementary material for: A phase II trial comparing pazopanib with doxorubicin as first-line treatment in elderly patients with metastatic or advanced soft tissue sarcoma (EPAZ): study protocol for a randomized controlled trial
Source: Trials. 2016 Jul 7;17:312. doi: 10.1186/s13063-016-1434-x (PMC4936293; doi:10.1186/s13063-016-1434-x)
Supplement: Additional file 3: — List of all ethical bodies that approved the EPAZ study. (DOCX 14 kb) [file 13063_2016_1434_MOESM3_ESM.docx]

**Additional File 3: List of all ethical bodies that approved the EPAZ study**

| **Germany** | |
| --- | --- |
| (leading ethics committee)  Ethikkommission der  Medizinischen Hochschule Hannover  Carl-Neuberg-Str. 1  30625 Hannover |  |
| Medizinische Ethikkommission II  Universitätsmedizin Mannheim  Maybachstraße 14-16  68169 Mannheim | Ethikkommission des  Fachbereichs Medizin  Pettenkoferstraße 8a  81336 München |
| Ethikkommission der  Universität Tübingen  Gartenstraße 47  72074 Tübingen | Ethikkommission der Med. Fakultät der  Universität Duisburg-Essen  Universitätsklinikum Essen  Robert-Koch-Straße 9-11  45147 Essen |
| Ethik-Kommission der med. Fakultät  der Technischen Universität  Fetscherstraße 74  01307 Dresden | Ethikkommission der Ärztekammer Hamburg  Humboldtstraße 67a  22083 Hamburg |
| Landesamt für Gesundheit und Soziales Berlin  Geschäftsstelle der Ethikkommission  des Landes Berlin  Fehrbelliner Platz 1  10707 Berlin | Ethikkommission der Med. Fakultät der  Universität Köln  Gebäude 5 Kerpener Straße 62  50937 Köln |
| Ethikkommission der Medizinischen Fakultät Heidelberg  Alte Glockengießerei 11/1  69115 Heidelberg | Ethik-Kommission der Med. Fakultät  Universitäts-Kinderklinik Arnold-Heller-Straße 3, Haus 9 24105 Kiel |
| Ethik-Kommission an der Medizinischen Fakultät der RWTH Aachen  Pauwelsstraße 30 52074 Aachen | Ethik-Kommission des Fachbereichs Medizin  der Goethe-Universität Theodor-Stern-Kai 7 60596 Frankfurt am Main |
| **Belgium** | |
| UZ Leuven campus Gasthuisberg Commissie medische ethiek Herestraat 49 3000 Leuven |  |
